# Predicting Response to Neoadjuvant Therapy in Colorectal Cancer Patients the Role of Messenger-and Micro-RNA Profiling

**DOI:** 10.3390/cancers12061652

**Published:** 2020-06-22

**Authors:** Alberto Izzotti, Chiara Ceccaroli, Marta Geretto, Filippo Grillo Ruggieri, Sara Schenone, Emilio Di Maria

**Affiliations:** 1 Department of Experimental Medicine, University of Genova, 16132 Genova, Italy; marta_geretto@hotmail.it; 2 IRCCS Ospedale Policlinico San Martino, 16132 Genova, Italy; chicec@hotmail.it; 3 Department of Radiotherapy, Galliera Hospital-Via, 16128 Genoa, Italy; filippo.grillo.ruggieri@galliera.it; 4 Department of Health Sciences, University of Genova, 16132 Genova, Italy; sara.schenone.16@gmail.com (S.S.); emilio.dimaria@unige.it (E.D.M.); 5 Unit of Medical Genetics, Galliera Hospital, 16128 Genoa, Italy

**Keywords:** colorectal neoplasms, neoadjuvant therapy, biomarkers, microRNA, miRNAs, RNA, messenger

## Abstract

Colorectal cancer patients’ responses to neoadjuvant therapy undergo broad inter-individual variations. The aim of this systematic review is to identify a molecular signature that is predictive of colon cancer downstaging and/or downgrading after neoadjuvant therapy. Among the hundreds analysed in the available studies, only 19 messenger-RNAs (mRNAs) and six micro-RNAs (miRNAs) were differentially expressed in responders versus non-responders in two or more independent studies. Therefore, a mRNA/miRNA signature can be designed accordingly, with limitations caused by the retrospective nature of these studies, the heterogeneity in study designs and the downgrading/downstaging assessment criteria. This signature can be proposed to tailor neoadjuvant therapy regimens on an individual basis.

## 1. Introduction

Colorectal cancer (CRC) represents the second most common cancer in the world, with an estimated number of prevalent cases (1-year) in 2018 of 1,356,151; the prevalence estimates for 2018 were computed using sex-, site-, and age-specific ratios of incidence to 1-year prevalence from Nordic countries for the period 2000–2009; these ratios were scaled using Human Development Index (HDI) ratios. The estimated number of incident cases worldwide (per year) is 1,849,518 [1].

The relative survival rate for CRC is 65% at 5 years after diagnosis and 58% at 10 years. The 5-year survival reaches 90% when the cancer is diagnosed at the localized stage of the disease, but this situation occurs only for 39% of CRC patients. When CRC is diagnosed in the regional stages, 5-year survival is reduced to 71%, while the reduction reaches up to 14% in patients in distant stages [2].

Rectal cancer comprises more than a third of cases [1]. Although less frequent than colon cancer, it seems to have many similar features to colon cancer in terms of geographic distribution. However, differences in some colon and rectal cancer risk factors reflect different patterns of genesis and development of rectal cancers with respect to the colon [3]. Rectal cancer is more difficult to cure than colon cancer due to a higher probability of spreading to other tissues, recurrence and postoperative complications. The diagnosis, staging and treatment regimens for rectal cancer differ from those for colon cancer [4]. The currently established standard of care for patients with locally advanced rectal cancer stage T3, stage T4, or node-positive disease (LARC) involves preoperative concurrent radiotherapy and chemotherapy, also known as neoadjuvant chemoradiotherapy (nCRT), followed by surgery. The main purpose of this therapeutic strategy is to reduce local recurrence. However, this treatment carries a significant risk of harmful side effects and has a highly variable response rate. Outcomes vary from the favourable “pathologic complete response” (pCR), which occurs in 10–20% of patients [5], with up to 30% of patients having no response to treatment [6].

Predictive biomarkers have been the subject of a great deal of studies with the aim of pretreatment risk stratification to determine the subset of patients who are expected to receive the most benefit and the least harm (responders). Conversely, non-responder patients could avoid receiving an ineffective therapy [7]. Accordingly, there is a strong need to modulate the nCRT regimens according to the predicted responder/non-responder status. Over the past 15 years, a major research effort has been directed at using genomic techniques to establish genomic and epigenomic signatures in order to assess prognosis. Since <5% of translationally activated genes are effectively translated into proteins [8], epigenomic signatures are potentially more predictive than genomic analyses. Furthermore, oncogene mutations are detectable only in a minority of cancer patients, while epigenomic alterations targeting microRNAs (miRNAs) are always present [9].

The microarray technique is a valuable tool to simultaneously profile thousands of messenger RNAs (mRNAs) or mature miRNAs in order to assess expression signatures for diagnosis, prognosis and therapeutic strategies. miRNAs are short, non-coding RNAs that play a pivotal role in regulation of gene expression. They act by inhibiting mRNA translation through recognition and binding to target sequences located within the mRNA itself. miRNAs are involved in many physiological functions, including cell proliferation, apoptosis and migration, both during development and adult life [10]. In cancer, miRNAs are often dysregulated, and thus miRNA expression analysis is a valid tool in diagnosis and therapy [9,11,12].

Although several mRNAs and miRNAs seem to have an impact on the response of colorectal cancer patients to nCRT, there is still only a small overlap and even conflicting results between different studies. Despite the genuine efforts of several groups of researchers in the field, there is a noteworthy lack of knowledge about the use of mRNAs and miRNAs as predictive biomarkers of neoadjuvant therapy in CRC.

The aim of the present work is to provide a comprehensive review of the literature, based on a systematic search, and to summarize the current data on mRNAs and miRNA expression as associated to colorectal cancer outcome and nCRT response, namely tumor downgrading and/or downstaging. These findings may shed new light on the perspective of mRNA and miRNA expression analysis as predictive biomarkers for colorectal cancer.

## 2. Methods

### 2.1. Protocol and Registration

The patient population (CRC patients), intervention (nCRT), comparison (different mRNA/miRNA signatures) and outcome (downgrading/downstaging yes or no) (PICO) framework was applied to design the protocol of the systematic review.

The review strategy and details were registered in the PROSPERO database (The International Prospective Register of Systematic Reviews) (record CRD42020136285). The registration number was requested on June 2019 [13]. At that time, no similar review was published or ongoing according to the PROSPERO database.

### 2.2. Literature Search

In order to identify all potentially eligible studies, we performed a comprehensive search on the National Library of Medicine’s PubMed online catalogue and the Cochrane Database of Systematic Reviews from inception up to 29 May 2018; an update was carried out on 3 January 2019.

We developed a search strategy and adapted it for each database, using a combination of the following keywords: colorectal cancer, rectal cancer, response, sensitivity, resistance, neoadjuvant, pretreatment, preoperative, chemoradiation, chemoradiotherapy, radiochemotherapy, chemotherapy, radiotherapy, gene expression, gene expression profile, gene expression profiling, microarray, array, polymerase chain reaction, genetic signature, transcriptomic, transcriptional, biomarkers, molecular markers, molecular response; colorectal neoplasms, gene expression, neoadjuvant therapy and chemoradiotherapy, applied as MeSH-terms. An overview of the full strategy with search strings is provided in the Supplementary Materials (Table S1). Unpublished and grey literature data were not sought.

### 2.3. Inclusion and Exclusion Criteria

Studies were considered eligible in the systematic review if they met the following criteria: (i) they provided information on colorectal cancer patients’ nCRT response, with tumor downgrading and downstaging being the primary outcomes of interest; (ii) they examined the role of gene expression analysis, as measured by levels of RNAs (mRNAs or miRNAs), in predicting treatment response; (iii) the analysis was performed on colorectal cancer specimens obtained before starting nCRT. Only original articles published in English in peer-reviewed journals were eligible for data extraction. Animal and in vitro studies were not included. Letters, comments, editorials, and case reports were also excluded.

### 2.4. Identification and Selection of Studies

One reviewer initially scanned primary titles and abstracts to select potential full text articles for further scrutiny. When the title and abstract check did not lead to a paper being rejected, the full text of the article was obtained and carefully reviewed for inclusion by two reviewers. Inclusion or exclusion of each study was determined by discussion and a consensus between the two. Any disagreement was resolved through discussion or after consultation with a third author. Article bibliographies were also inspected in order to identify any additional studies that may have been missed during the initial search. Sponsors were not contacted. Authors were contacted once in order to retrieve the Supplementary Materials.

**Figure 1:**
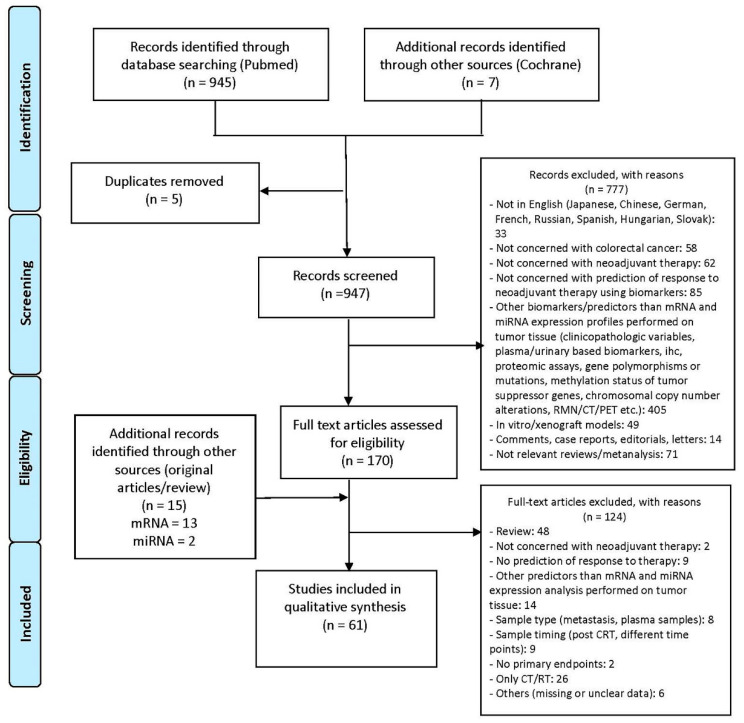
Flow chart reporting the procedure used to perform the systematic review.

The literature retrieval and selection procedures were in adherence to the Preferred Reporting Items for Systematic Reviews and Meta-Analyses (PRISMA) Statement. A flow diagram of the procedures is shown in Figure 1.

### 2.5. Data Extraction and Synthesis

Genes (mRNA) and miRNA expression data were extracted from the manuscript and Supplementary Materials of the included studies. The following data were sought for each miRNA/mRNA and recorded in excel tables: gene symbol and miRNA symbol; references; endpoints (primaries and others); definition of responders; differentially relative expression (in responders versus non-responders); availability of quantitative data (e.g., fold change); *p* value; notes.

The primary excel table was reordered by mRNA/miRNA species. The number of studies reporting association for each mRNA/miRNA was recorded. Relevant studies were considered if the *p*-value associated to this differential relative expression was 0.05 or less and the differential relative expression was related to the primary outcomes (downgrading and/or downstaging). Finally, top hits were selected if at least two studies reported a concordant differential relative expression (mRNA: increased or decreased expression; miRNA: up or downregulation). mRNA and miRNA tables are provided in the Supplementary Materials (Table S2—List of mRNAs differentially expressed in responders versus non responders and Table S3—List of miRNAs differentially expressed in responders versus non responders).

The relationship between miRNAs and their target mRNAs has been evaluated based on TargetScan total context score. The relationship between mRNA, miRNA and common oncogene mutations occurring in colorectal cancer was analysed using the Cosmic catalogue [14].

## 3. Results

### 3.1. Literature Search

A total of 945 articles were retrieved by a literature search of PubMed database and an additional seven articles were identified through the Cochrane Library. After removing duplicates citations (*n* = 5), the initial database searches identified 948 articles, which were screened based on their title and abstract. In total, 777 articles were removed for the following reasons: they were written in a language other than English (*n* = 33); they were not concerned with colorectal cancer (*n* = 58) or with neoadjuvant therapy (*n* = 62) or with prediction of response to neoadjuvant therapy using biomarkers (*n* = 85); they investigated biomarkers or predictors other than mRNA and miRNA in tumor tissue (*n* = 405); they were animal or in vitro studies (*n* = 49); they were not original articles (*n* = 14) or not relevant reviews/meta-analyses focused on the treated topic (*n* = 71). By manually screening the reference lists of the remaining 170 articles and relevant reviews, 15 additional records were identified and included. Of all the potentially 185 relevant full-text articles, 48 were removed because they were reviews or meta-analyses not reporting original data, two did not refer to neoadjuvant therapy, nine were not concerned with the prediction of responses to treatment and 14 investigated something other than mRNA or miRNA expression analysis in tumor tissue. Studies performed on samples other than primary tumor tissue (*n* = 8) or not obtained before the administration of neoadjuvant treatment (*n* = 9) were excluded, such as studies with endpoints different from primary ones (*n* = 2). Only studies including patients receiving a combined regimen of chemotherapy (CT) and radiotherapy (RT) were considered eligible, while studies in which treatment was either CT or RT alone were excluded (*n* = 26). One publication was not an original article (book chapter) and therefore was not considered for analysis. In total, 95 articles were removed from the search pool and the remaining 61 articles were included in this review.

The relationship between miRNAs and their target mRNAs has been evaluated based on TargetScan total context score and listed in Table 1.

The relationship between mRNA, miRNA and common oncogene mutations occurring in colorectal cancer was analysed using the Cosmic catalogue [14] (Table 2).

### 3.2. Analysis of mRNA Expression Data

The systematic literature analysis identified 674 genes holding predictive value. The list of all these genes is reported in Supplementary Materials, Table S2.

A total of 19 mRNAs were differentially expressed in two or more independent studies with a *p* value ≤ 0.05: BIRC5, CDV3, EGFR, EIF4A1, IPTK1, KCNJ2, LGR5, MMP14, MKI67, MT-ND4, MT-ND6, MYC, NME2, RRM1, STK11, TOP1, TYMP, TYMS, VEGFA.

Expression was concordant for 15 of them: *BIRC5, CDV3, EGRF, IPTK1, KCNJ2, LGR5, MT-ND4, MT-ND6, MYC, NME2, RRM1, STK11, TYMS, TYMP, VEGFA*. Functions and biological role of these genes, and other details, are reported below according to annotated ontology available on Gene cards, human gene database [15].

#### 3.2.1. Genes Modified in Two or More Studies

***(1)***  ***BIRC5*** encodes negative regulatory proteins that prevent apoptotic cell death. Its differentially relative expression (responder (R) versus non-responders (NR)) was analysed in three studies. In two of them, it was decreased in nCRT responders [16,17]. In one study, it was increased [18].
***(2)***  ***CDV3***—no additional information on Carnitine Deficiency-Associated Gene Expressed in Ventricle 3 is present on GeneCards. Its differentially relative expression (R versus NR) was analysed in two studies. In both, it was increased in nCRT responders [19,20].
***(3)***  ***EGFR*** encodes the protein Epidermal Growth Factor Receptor, a receptor for members of the epidermal growth factor family which binding leads to cell proliferation. Its differentially relative expression (R versus NR) was analysed in two studies. In both, it was decreased in nCRT responders [21,22].
***(4)***  ***(ITPK1*** encodes the protein Inositol-Tetrakiphosphate 1-Kinase, an enzyme regulating the synthesis of inositol tetraphosphate and downstream products. Inositol metabolism plays a role in the development of the neural tube and maintenance of histone gene-suppression function. Its differentially relative expression (R versus NR) was analysed in three studies. In two of them, it was increased in nCRT responders [23,24]. In one study, it was decreased, but only when associated with downsizing [25].
***(5)***  ***KCNJ2*** encodes Potassium Inwardly Rectifying Channel Subfamily J Member 5, a subunit of the homotetrameric potassium channel. Its differentially relative expression (R versus NR) was analysed in two studies. In both, it was increased in nCRT responders [19,26].
***(6)***  ***LGR5*** encodes Leucine Rich Repeat Containing G Protein-Coupled Receptor 5, a receptor involved in the Wnt signalling pathway; it also plays a role in the formation and maintenance of adult intestinal stem cells during postembryonic development. Associated diseases include colon adenoma. Its differentially relative expression (R versus NR) was analysed in two studies. In both, it was decreased in nCRT responders [22,27].
***(7)***  ***MT-ND4*** encodes Mitochondrially Encoded NADH Dehydrogenase 4 protein, involved in many pathways, such as respiratory electron transport, ATP synthesis, heat production by uncoupling proteins and GABAergic synapse. Its differentially relative expression (R versus NR) was analysed in two studies. In both, it was increased in nCRT responders [23,24].
***(8)***  ***MT-ND6*** encodes Mitochondrially Encoded NADH Dehydrogenase 6 protein, involved in the MT-ND4 pathways. Its differentially relative expression (R versus NR) was analysed in two studies. In both, it was increased in nCRT responders [23,24].
***(9)***  ***MYC*** is a proto-oncogene, key regulator of cell cycle progression, apoptosis and cellular transformation. Its differentially relative expression (R versus NR) was analysed in three studies. In all of them, it was increased in nCRT responders [20,28].
***(10)***  ***NME2*** encodes for a protein involved in the pathway of pyrimidine deoxyribonucleotides de novo biosynthesis and in the innate immune system pathway. Its differentially relative expression (R versus NR) was analysed in two studies. In both, it was increased in nCRT responders [20,29].
***(11)***  ***RRM1*** encodes the protein Ribonucleotide Reductase Catalytic Subunit M1, subunit of ribonucleotide reductase, an enzyme essential for the conversion of ribonucleotides into deoxyribonucleotides, which are important for DNA replication and repair. Its differentially relative expression (R versus NR) was analysed in two studies. In both, it was increased in nCRT responders [20,29].
***(12)***  ***STK11*** encodes the protein Serine/Threonine Kinase 11, a tumor suppressor; mutations in this gene have been associated with Peutz–Jeghers syndrome. Its differentially relative expression (R versus NR) was analysed in two studies. In both, it was decreased in nCRT responders [22,27].
***(13)***  ***TYMP*** encodes the protein Pyrimidine Metabolism Enzyme Thymidine Phosphorylase, an angiogenic factor that promotes angiogenesis in vivo and stimulates the in vitro growth of endothelial cells. Its differentially relative expression (R versus NR) was analysed in five studies. In four of them, it was increased in nCRT responders [30,31,32,33]. In one study, it was decreased, but only when associated with no distant recurrence [34].
***(14)***  ***TYMS*** encodes thymidylate synthase, an enzyme responsible for DNA methylation, playing a pivotal role in DNA replication and repair. Its differentially relative expression (R versus NR) was analysed in eight studies and was decreased in nCRT responders [14,32,34,35,36,37,38]. In particular, nCRT response was reported always in terms of tumor regression grade (according to various classification) in all eight studies. Moreover, nCRT response was also reported as downstaging in two studies [14,35] and as disease-free survival in another two studies [34,35]. Another studies [39] reported nCRT response in terms of disease specific survival and recurrence free survival; therefore, it was excluded in the systematic analysis of all genes differentially expressed.
***(15)***  ***VEGFA*** encodes Vascular Endothelial Growth Factor A, a growth factor protein inducing the proliferation and migration of vascular endothelial cells. Its differentially relative expression (R versus NR) was analysed in three studies. In all of them, it was decreased in nCRT responders [21,32,40].

#### 3.2.2. Main Biological Function of other Genes Identified as nCRT Predictors

A full list of these genes is reported in Supplementary Materials, Table S2. Below are reported and discussed only pivotal examples of genes as belonging to each biological function examined.

***Apoptosis***—the following mRNAs were upregulated in nCRT responders: *BAX* (encoding for a protein which promotes apoptosis by binding apoptosis repressor Bcl2) [19,23], *CFLAR* (encoding for a protein structurally similar to caspase 8, although it lacks caspase activity, and acting as an apoptosis regulator by inhibiting TNFRSF6) [20], *ETS2* (transcription factors involved in development and apoptosis through transcriptional activation) [19], *GGCT* (encoding for Gamma-Glutamylcyclotransferase, an enzyme that plays a pivotal role in glutathione homeostasis and that induces cytochrome c release from mitochondria, leading to apoptosis induction) [20], *ID1* (encoding for a protein that negatively regulates helix–loop–helix transcription factors through heterodimer formation and consequently inhibition of their transcriptional activity) [20], *SEPTIN7P2* (encoding for a protein involved in the recruitment of other proteins) [41] and *TRIAP1* (encoding for a protein involved in modulation of mitochondrial apoptotic pathway through inhibition of caspase-9) [20]. Two genes were downregulated in responders, including *BAK1* (encoding for a protein inducing apoptosis through accelerating the opening of the mitochondrial voltage-dependent anion channel, which leads to a loss in membrane potential and the release of cytochrome c; it also interacts with the tumor suppressor P53 after exposure to cell stress) [38] and *SHF* (encoding for a protein playing a role in the regulation of apoptosis in response to PDGF(Platelet Derived Growth Factor)) [41]. Among genes related to programmed cell death, *MTCH1* [42], *PYCARD* [36] and *TRAF4* [36] were identified as part of a gene set correlated with downgrading.

***Cell cycle***—*PPP1R10*, encoding for a protein phosphatase involved in cell cycle progression, DNA repair and apoptosis, is part of a gene signature related to tumor downsizing after nCRT [25]. In addition, a follow-up study conducted on the same cohort of patients demonstrated that the previously identified set of genes was correlated also with disease free survival [43]. Several genes involved in cell cycle regulation are characterized by different expression levels between responders and non-responders to nCRT. mRNA levels of *CCNK* (encoding for Cyclin K, which regulates transcription through phosphorylation of the C-terminal domain of the large subunit of RNA polymerase II) [19], *RIF1* (encoding for a protein that shares homology with the yeast telomere binding protein, Rap1 interacting factor 1, localizing to aberrant telomeres may be involved in DNA repair) [19], *TCF19* (encoding for a protein containing a PHD-type zinc finger domain and likely functioning as a transcription factor, playing a role proliferation and apoptosis of pancreatic beta cells) [41] and *TOE1* (encoding for target of EGR1 Protein 1, which inhibits cell cycle progression and consequently arrests cell growth rate) [19] were found to be over-expressed in responders. Conversely, *SPDYA* (encoding for a protein that regulates the G1/S phase transition) were part of a set of 57 genes found downregulated in nCRT responders [41]. *TP53* (tumor suppressor protein) was also downregulated in responders to nCRT [32,42,44].

***Inflammatory response***—Palma et al. reported in CRC patients responding to nCRT upregulation of the *CXCL3* gene that encodes for chemokine ligand 3 and takes part in inflammation through its neutrophil chemotactic activity [20].

***Immune response****—AKIRIN2* and *DRB1* are both involved in immune response and were part of a 257-gene signature that showed upregulation in responders [20]. *AKIRIN2* is required for innate immune response and represents a downstream effector of Toll-like receptor, TNF and IL-1(Interleukin-1) beta signalling pathways, being therefore responsible for IL-6 production. *DRB1* encodes for HLA class II molecules beta chain, located on the surface of antigen-presenting cells. The main role of these cells is to bind peptides derived from antigens and presenting them on the cell surface in order to trigger the immune response. nCRT responders have lower *BTNL8* expression as compared to nCRT non-responders in CRC biopsies collected before treatment as related to tegafur/gimeracil/oteracil and tegafur/uracil nCRT regimens [45]. Butyrophilin Like 8 (BTNL8) stimulates innate immune response leading to T-cell proliferation and cytokine production.

### 3.3. Analysis of miRNA Expression Data

The systematic literature analysis identified 77 miRNA holding predictive value. A list of all 77 miRNAs is reported in the Supplementary Materials, Table S3.

A total of six miRNAs were found to be differentially expressed in two or more independent studies with a *p* value ≤ 0.05: let-7f, miR-21, miR-145, miR-622, miR-630 and miR-1183 (Table 1).

#### 3.3.1. Upregulated miRNA in Responders

In particular, as reported by Nakao et al. (2015), miR-31, miR-34b, miR-144, miR-154, miR-193a, miR-223, miR-335, miR-363, miR-379, miR-382, miR-451, miR-486, miR-542, miR-1246, and miR-1290 were characterized by an increased expression level in complete and partial nCRT responders (RECIST guidelines) treated with chemo-radiotherapy consisting of tegafur-uracil combined with a total of 40 Gy radiations. The same observation applies to miR-19, miR-494, miR-513a, miR-513b, miR-866 and miR-923 that were upregulated in complete and partial responders treated with tegafur/gimeracil/oteracil combined with a total of 40 Gy radiation [45].

Considering some of these miRNAs, another study found, instead, that miR-31 overexpression was related to poor pathological nCRT response while patients characterized by miR-31 downregulation generally showed a better nCRT response [46]. Regarding miR-451, another study reported a different result. Indeed, this miRNA was downregulated in responders TRG 1 and TRG 2 patients [28] (downgrading according to Mandard’s tumor regression grading system – TRG).

In Hotchi et al., miR-126, miR-142, miR-223 and miR-630 upregulation was significantly correlated with nCRT response [47].

miR-125a, miR-188, miR-483, miR-622, miR-630, miR-671, miR-765, miR-1183, miR-1224, miR-1471, and miR-1909 upregulated expression was correlated with TRG 1 tumor downgrading according to Mandard et al. [48].

Ma et al. found a different result for miR-622. Indeed, in their study they showed that miR-622 downregulation correlated with TRG 1–3 downgrading according to Mandard et al. [49].

A different result was also reported for miR-1183. Indeed, this miRNA was downregulated in TRG 1 and TRG 2 responders (downgrading according to Mandard et al.) [28].

In Svoboda et al., TRG 1–3 downgrading (according to Mandard et al., modified) showed a correlation with miR-99a, miR-196b, miR-450a, miR-450b and let-7e upregulation [50].

The same trend has been reported by Lopes Ramos et al. for miR-21, miR-1246 and miR-1290, which were upregulated in responders (TRG 4, downgrading according to Dworak et al.) [23]. miR-21 upregulation was also reported by Eriksen et al., who found a significant association between high miRNA-21 expression and TRG 1 and TRG 2 responders [51].

In both Findlay and Drebber et al., miR-145 was upregulated in responders with TRG 3–4 (downgrading, respectively, according to Rodel et al. and to Schneider et al.) [52,53]. A correlation between TRG 2–3 downgrading (according to Japanese Society for Cancer of the Colon and Rectum) and miR-142 and miR-223 upregulation was found by Hotchi et al. [47]. Regarding miR-145, Eriksen et al. and Conde-Muino et al. identified a significant association between low miRNA-145 expression and TRG 1 and TRG 2 responders [28,51].

Among the most recent studies found in literature, D’Angelo et al. reported that TRG 1–2 responders (downgrading according to Mandard et al.) were characterized by miR-194 overexpression [54] and Conde-Muino et al. described miR-18a upregulation in responders characterized by the same Mandard’s score [28]. In another study performed by D’Angelo, miR-154, miR-409, miR-127, miR-214, miR-299, miR-125b were upregulated in non-responders to nCRT [55]. Finally, Yu et al. found that miR-92b, miR-141 and miR-6776 were upregulated in TRG1 patients (downgrading according to Mandard et al.) and were part of a miRNA signature [56].

#### 3.3.2. Downregulated miRNA in Responders

miR-190b, miR-215, miR-29b-2 were downregulated in nCRT responders in terms of TRG (TRG 1–3, downgrading according to Mandard et al., modified) [50], as well as miR-1274b and miR-720, which showed the same trend in TRG 1 tumors (downgrading according to Mandard et al.) [48], miR-205 in TRG 4 tumors (downgrading according to Dworak et al.) [23], and miR-21 in TRG 0 tumors (downgrading according to Ryan et al.) [57].

Kheirelseid et al. reported a complete response in terms of tumor downgrading (tumor regression grade by Mandard et al.) according to the following miRNA signature: miR-16, miR-153 and miR-590. Moreover, miR-519c and miR-561 showed a significant correlation with a good nCRT response [58] (it was not reported whether up- or downregulated).

Yu et al. found that miR-345, miR-1180, miR-1281, miR-4433b and miR-5739 were part of a signature and were downregulated in TRG 1 patients (downgrading according to Mandard et al.). In particular, authors focused their attention on miR-345, showing that the expression of this miRNA is significantly lower in nCRT responders as compared to nCRT-resistant patients [56]. Conde-Muino et al. found that a signature composed of miR-30b, miR-148a, miR-375, miR-519b, miR-650, miR-1233, miR-1243 and let-7f were downregulated in TRG 1 and TRG 2 responders (downgrading according to Mandard et al.) [28].

Finally, D’Angelo et al. found that miR-33a, miR-30e, miR-338, miR-200a e miR-378 were downregulated in non-responder to nCRT [55].

## 4. Discussion

The presented results provide evidence that several epigenetic biomarkers, both mRNA and miRNA, have been identified as predictors of nCRT response in at least one study. However, none of these biomarkers appear to be predictive when examined alone. Furthermore, significant discrepancies exist between different studies. To be predictive, an association has to be supported by a plausible biological mechanism. Accordingly, we examined the possible mechanism supporting the association between biomarker and nCRT response.

In relation to mRNA, the genes whose expression have a major role in predicting nCRT response are *TYMS*, *TYMP, ITPK1*, *MKY76*, *MYC* and *TOP1*.

The augmented responsiveness to neoadjuvant therapy in tumors with elevated expression of *MYC* could be mediated by its “growth promoting/death priming” action [54,59]. In fact, an increase in *MYC* expression facilitates incorporation of 5-FU (5-Fluoruracil) into the S phase of the cell cycle, thus increasing cell susceptibility to this compound [28]. However, Palma et al. detailed that only *MYC* mRNA expression levels correlates with nCRT response; conversely, no correlation exists with MYC gene amplification and *MYC* protein overexpression [20].

Other predictor mRNAs are related to the expression of gene involved in apoptosis, cell cycle, inflammation and immune response, modulation of epidermal growth factors, hypoxia-inducible factors and vascular endothelial growth factors, as discussed below.

***Cell cycle***—the cell cycle is fundamental in conditioning cancer cell susceptibility to nCRT, with proliferating cells being much more sensitive than quiescent cells. As reported by Vignard et al., the *RIF1* pathway is involved in the DNA repair of damage induced by ionizing radiation [60], this finding making plausible its role in nCRT response.

***Inflammation and immune response***—inflammation plays a pivotal role in modulating the radiation responsiveness of tumors. Radiation therapy represents a dual-edged sword treatment modality for many cancers. Furthermore, to kill cells by genotoxic damage, ionizing radiation (IR) triggers, in surviving cancer cells, a nuclear DNA damage response, inducing the transcription of pro-inflammatory factors and promoting resistance to radiation and cancer development. Among them, *NF-kB*, *STAT3* and *HIF1* play a crucial role in the radiation-induced inflammatory response [61]. Together, these transcription factors regulate a wide spectrum of genes involved in inflammation, apoptosis, invasion and angiogenesis, contributing to confer radio-resistance to cancer cell. A number of studies confirm that selective inhibitors of these pro-inflammatory pathways could be associated to conventional radiation or chemotherapy [62]. However, there is increasing evidence that radiotherapy supports tumor-specific immunity, given that radiation damage creates a link between inflammation and immune activation [63]. Indeed, radiotherapy leads to significant alterations in the tumor microenvironment, particularly with respect to immune cells infiltrating tumors [64]. Chakraborty et al. demonstrated that radiotherapy influenced the tumor site, rendering cancer cells more susceptible to T cell-mediated cytotoxicity, through the upregulation of a range of adhesion molecules [65]. Radiation increases the expression of major histocompatibility complex class I; indeed, accentuating this effect via gene therapy increases the therapeutic efficacy of radiation. Tumour antigen-specific cells elicited by radiation can upregulate IFN-γ (Interferon gamma) in the tumor, and responsiveness to IFN-γ has been shown to be required for radiation-induced major histocompatibility complex upregulation. The combination of radiation-induced local inflammation and tumor-specific effector T cells can, together, alter the tumor vasculature, providing an additional mechanism of tumor control [66]. Recent data suggest that irradiation with a few radiotherapy fractions at high doses, with a break between the fractions (a “radiation holiday”), results in the activation of immunity, protecting against cancer progression. Conversely, standard hyper-fractioned radiotherapy, given as daily low-dose fractions during a prolonged period, results in chronic inflammation, which is immunosuppressive, supporting angiogenesis and cancer progression [67,68]. Inflammatory response is a typical feature of the tumor microenvironment consisting of cellular components (cancer cells, lymphocytes, endothelial cells, fibroblasts and tumor-associated macrophages (TAMs)) and signalling molecules (chemokines and cytokines). In many cancer types, TAMs are associated with poor prognosis, while in CRC their role is still controversial. As reported by Erreni et al. [69], TAMs’ anti-tumor or pro-tumor activity depends on their localization in cancer tissue. Peritumoral TAMs preferentially differentiate into the tumoricidal phenotype because they are less exposed to tumor-derived cytokines. Takano et al. showed that the differentiation of monocytes to M2-TAMs was determined by miR-203. Indeed, circulating exosomes carrying miR-203 are released by CRC cells and are incorporated into monocytes, leading to their differentiation to TAMs. miR-203 promotes M2 differentiation by downregulating *suppressor of cytokine signalling 3* (*SOCS3*) [70]. This mechanism is responsible for the formation of premetastatic niches, as reported in pancreas and breast cancer [71,72]. Moreover, as reported by Yin et al., TAMs are involved in CRC chemoresistance through IL-6 secretion and IL-6R/STAT3/miR-204-5p activation pathway [73]. *AKIRIN* and *DRB1* mRNAs are upregulated in nCRT responders [20]. *AKIRIN* is required for innate immune response and represents a downstream effector of Toll-like receptor, TNF and IL-1 beta and IL-6. *DRB1* encodes for the HLA class II beta chain, located on the surface of antigen-presenting cells. Butyrophilin Like 8 (*BTNL8*) mRNA is down regulated in nCRT respondres [45]. *BTNL8* is involved in innate immune response, leading to T-cell proliferation and cytokine production.

***Epidermal Growth Factor modulation***—several genes involved in epidermal growth factor modulation are targeted by different miRNAs, including let-7g, miR-145, miR-223, miR-622, miR-630 and miR-1246. In the studies included in the present review, patients responding to nCRT showed higher levels compared to non-responders of all these *EGFR* regulating miRNAs. This miRNA upregulation resulted in the downregulation of EGFR expression in patients with significant tumor regression [21,27]. These miRNAs are also involved in silencing the expression of EGFR oncogenic mutations. Metzger et al., sequencing the DNA of 236 CRC tumor samples from European patients, found somatic missense mutations in *EGFR* exons 18 and 20 at very low frequency, 2.1% and 0.4%, respectively [74]. A study on Korean CRC patients showed that the incidence of EGFR mutation is 22.41% and all these mutations were detected in exon 20 [75]. Some studies focused on tumor *EGFR* copy number in relation to monoclonal antibody therapies resistance. Indeed, as reported by Moroni et al., colorectal cancer characterized by an increased *EGFR* copy number, presents a higher sensitivity to cetuximab and panitumumab based therapies. Moreover, this therapeutic strategy is not effective in tumors with a low *EGFR* copy number [76,77]. However, no study has thus far analysed both miRNA and *EGFR* mutations in parallel in the same patients.

***Hypoxia-inducible factor modulation***—the cancer microenvironment is characterized by a remarkable hypoxic condition. When a reduced oxygen supply occurs, *HIF*s are mainly responsible for changes in gene expression. *HIF-1* is considered a key regulator in oxygen homeostasis [78]. *HIF-1* is responsible for activation of genes involved in energy metabolism, angiogenesis, and apoptosis. Moreover, this transcription factor plays an essential role in tumor angiogenesis [15]. Talks et al. found that *HIF1A* and *HIF2A* were expressed in bladder, brain, breast, colon, ovarian, pancreatic, prostate, and renal carcinomas. Moreover, *HIF2A* was highly expressed by TAMs. On the other hand, *HIF1A* and *HIF2A* were not found in healthy tissues except for bone marrow macrophages, where *HIF2A* was highly expressed [79]. In different cancer types, such as breast cancer, colorectal cancer and esophageal carcinoma, *HIF-1* is involved in different steps of metastatization process, such as epithelial-mesenchymal transition (EMT), invasion and formation of metastatic niche [80,81,82]. *HIF1A* is targeted by two specific miRNAs, miR-622 and miR-630 [83]. These miRNAs are upregulated in nCRT responders, a situation paralleled by a reduction in *HIF1A* [48]. The same correlation with tumor downgrading was found for miR-145 targeting *HIF*, which is upregulated in nCRT responders [52,53]. let-7g is a *HIF1A* inhibitor, its upregulation causing a decrease in *HIF1A* expression. Since *HIF1A* is involved in cellular metabolism and in angiogenesis, its decreased expression explains, at least in part, the tumor regression observed in responders [84]. These miRNAs are also involved in silencing the expression of HIF oncogenic mutations. Only a few studies regarding *HIF1A* gene mutations and CRC have been carried out. Two common single nucleotide polymorphisms (SNPs) within the oxygen-dependent degradation domain of the *HIF1A* gene have been described: P582S (rs115494650; C1772T) and A588T (rs11549467; G1790A). These SNPs are associated with CRC susceptibility as well as clinical and pathological features. Indeed, patients bearing the 588T allele showed a larger tumor size than wild type carriers [85]. Sometimes, detected mutations do not affect the gene itself but a gene located upstream in the cascade, as reported by Kuwai et al. (2004). These authors identified that 53.8% of von Hippel–Lindau (VHL) gene mutants were associated with an increased *HIF1A* expression, as demonstrated by immunohistochemical analysis [86]. However, no study has thus far analysed both miRNA and *HIF* mutations in parallel in the same patients.

***Vascular endothelial growth factor modulation***—*VEGF* expression is increased in many tumors and correlates with tumor staging and progression [87]. *VEGFA* is targeted by miR-622. miR-622 is upregulated in nCRT responders [43] with a parallel decrease in *VEGFA* expression leading to a decreased endothelial cell proliferation with a consequent reduction in new blood vessel formation, feeding cancer mass [21,40]. According to the TargetScan database, *VEGFB* is targeted by miR-622 and *VEGFC* is targeted by miR-145-5p, miR-223-5p, and miR-630 [83]. Vlajnic et al. reported that the *VEGFA* gene locus is amplified in a subgroup of patients (3–6%) with CRC. This amplification is associated with highly aggressive CRC, poor prognosis and reduced survival time [88]. Furthermore, Burmeister et al., studying CRC immune microenvironment, found that *VEGFA* gene amplification is related to M1/M2 macrophage reduction and the decreased expression of PD-1 in lymphocyte-infiltrating cancer tissue, leading to a weakened immune response against cancer cells [89]. These findings are of relevance for new-targeted CRC therapies involving the use of PD-1 inhibitors. Indeed, as reported by Overman et al. [90], anti-PD-1 monoclonal antibody (Nivolumab) has shown its effectiveness in patients bearing metastatic CRC characterized by DNA-mismatch repair-deficiency or microsatellite instability. In 2017, the FDA (Federal Drug Administration) approved Nivolumab-based therapy for these patients. *VEGF* expression is closely related to *HIF1A*. Indeed, *HIF1A* binds to hypoxia responsive elements promoter region of *VEGF* leading to its activation. This activation is essential for CRC angiogenesis demonstrating that *HIF1A* and *VEGF* are key molecules involved in tumoral blood vessel formation [91]. In particular, *VEGFA* is produced by TAMs in response to hypoxia in avascular areas of tumors [92] and is responsible for macrophage progenitors’ recruitment before their differentiation to TAMs under IL-4 influence [93].

### 4.1. miRNAs Differential Expression

In included studies, downregulated miRNAs represent about a quarter of all miRNAs differentially expressed in responders. Most of them, including let-7a, miR-17, miR-20a, miR-20b, miR-92a, miR-106a, and miR-181b showed a significant correlation only for tumor downsizing without any connection to downstaging and downgrading [47,84].

Among the mentioned miRNAs, miR-16 and miR-451 are known for their role in resistance to chemotherapy [94]. miR-16 expression is reduced in gastric, breast and lung cancer cell lines contributing to tumor resistance to Vincristine, Tamoxifen and Plumbagin, respectively. Tumour resistance can be overcome by transfecting miR-16 into the cells [95,96,97,98]. In ovarian and cervical cancer, miR-451 is implicated in Doxorubicin and Vinblastine resistance, as reported by Zhu et al. The main target of this miRNA is represented by P-glycoprotein, a member of the ATP-binding cassette family, which is located in the cancer cell membrane and is responsible for increased drug efflux [91]. Another miRNA involved in tumor chemoresistance is miR-125a: its upregulation in human pancreatic cancer cell line SW1990 induces chemoresistance to gemcitabine, while its downregulation restores drug sensitivity [99].

However, to classify a miRNA as predictive of nCRT response, its targets, including modulated protein and/or suppressed oncogenic mutation, also have to be involved in nCRT response.

### 4.2. Identification of Targeted Proteins

The predictive potential of mRNA and miRNA is related to their chance of affecting protein synthesis. This chance is very limited for mRNA, with only less than 5% of them being effectively translated into proteins, with miRNA influence being much more consistent^8^. Accordingly, it is important to evaluate the correlation between mRNA/miRNA signatures with those of proteins identified as predictive of nCRT response.

For each protein, the corresponding mRNA/miRNA was checked among those identified as predictive of nCRT response (Table 2).

In order to identify changes in protein expression profiles among CRC patients, Hao et al. performed a proteomic analysis both on 22 CRC-paired tumors and adjacent normal tissues using mass spectrometry [100]. Proteins involved in cell proliferation, such as cyclin-dependent kinases (CDKs) and mitosis factors showed an increased expression in tumoral tissue. These findings are in agreement with the role of *CDK10* gene expression as predictor of nCRT response [101] and the many miRNA involved in its regulation (see Table 1).

Moreover, Hao et al. reported, in CRC tissues, an increased expression of the proteins responsible for DNA damage repair, including nucleotide excision repair (NER), base excision repair (BER), homologous and non-homologous end joining. An analysis of enzymes involved in aerobic glycolysis pointed out an increased expression of LDH-A (lactate dehydrogenase), pyruvate kinase, fructose-2, 6-bisphosphatase, and glucose-6-phosphate 1-dehydrogenase as well as proteins involved in lipid metabolism. Among proteins that undergo a decreased expression in tumor tissue compared to adjacent normal mucosa, the authors identified extracellular matrix (ECM) components. Conversely, the expression rate of matrix metalloproteinases was increased, suggesting a progression towards metastasis development [100].

Yao et al., using SDS (Sodium Dodecyl Sulphate) polyacrylamide gel electrophoresis (PAGE) and an electrospray ionization ion trap mass spectrometer, found that (Epidermal Growth Factor) EGF-containing fibulin-like extracellular matrix protein 2 (EFEMP2) was highly upregulated in CRC patients. Results were validated using both immunohistochemistry and ELISA assay [99]. Altered expression of membrane proteins in several CRC samples was found using the 2D-DIGE technique. Among these proteins, annexin A4 and voltage-dependent anion channel (VDAC) were proposed by the authors as promising biomarkers for CRC diagnosis and response to therapy [102].

Zhao et al., performing 2-D electrophoresis and Western blot analysis in metastatic CRC samples, pointed out that Rho GDP-dissociation inhibitor was upregulated, correlating with tumor invasion [103].

Bowden et al., using tissue microarray staining for acid ceramidase in CRC cells from diagnostic biopsy samples, normal adjacent colon and resection specimens, found that a higher expression of acid ceramidase was associated with a poor nCRT response (the results were statistically insignificant for the diagnostic biopsy samples). Acid ceramidase is a sphingolipid and catalyses the cleavage of ceramide, a lipid that has been observed to accumulate in radiation-induced apoptosis. Thus, high levels of acid ceramidase decrease the tumor suppressor activity of ceramide [104].

Chauvin et al. analysed the proteome of CRC cells from diagnostic biopsy samples. According to the American Joint Committee on Cancer (AJCC) tumor regression grading, they found that the levels of eight proteins (DPYD, CALD1, CPA3, B3GALT5, CNNM4, MTIF3, CD177 and RIPK1) were higher in TRG 3 patients, whilst the levels of other different 31 proteins (TYMP, PVR, IFIT1, F12, FASTKD2, BPGM, PIP4K2B, MCMBP, CHD1L, ENSA, PVRL1, TRMT5, CNOT10, SLC25A33, FTO, AMDHD2, PHPT1, SLC5A6, LSM12, TSPAN6, CBX1, NOSIP, TSSC4, ARID1B, ALDH3A1, AAMDC, GTF2E1, SNX7, STX10, ABLIM1, ERBB2) were higher in TRG 0 patients; moreover, subtypes of ribosomal and mitochondrial proteins were better represented in TRG 3 patients. Among all these proteins, DPYD, TYMP and ARID1B were involved in CRC pathways. DPYD, overexpressed in TRG 3 patients, is an enzyme responsible for approximately 80% of the hepatic elimination of 5-FU. TYMP (also called Platelet-Derived Endothelial Cell Growth Factor (PD-ECGF)), overexpressed in TRG 0 patients, has both a tumorigenic role (stimulating metastasis, invasion, angiogenesis and cell death invasion) and a 5-FU sensitizer role. Ribosomal (in particular, those composing the small and the large subunit of the ribosome) and mitochondrial proteins were more represented in TRG 0 patients, probably because of the action of 5-FU on pre-rRNA maturation and mitochondrial ribosome biogenesis and/or tRNAs, respectively [105]. Besides, Repetto et al. did not find the same proteins as predictive of response in CRT [106].

Finally, Ma et al. focused on the protein produced by the tumor suppressor retinoblastoma gene (RB1), a miR-622 target gene related to apoptosis and cell cycle. Both in pretreatment CRC cells of TRG4 patients and in post-treatment radiation-resistant CRC cells, an analysis of miRNAs expression revealed that miR-622 expression is significantly higher compared, respectively, to pretreatment CRC cells of TRG1–3 patients and post-treatment radiation-sensitive CRC cells. Moreover, when the latter were transfected with miR-622, Western blotting assays with antibodies targeting RB1 showed a reduction in this tumor suppressor level. Conversely, when Rb was transfected into miR-622-containing cells, radioresistance decreased. Interestingly, the expression of miR-622 increases in a radiation dose-dependent manner, both in single-dose radiation-treated cells (8 Gy for once) and in continuous low-dose radiation-treated cells (2 Gy/day for 7 days) [49].

### 4.3. Correlation of miRNA with Suppressed Oncogenic Mutation

Several mutations in genes involved in different cellular pathways have been discovered in CRC. According to the Cosmic database [14], *TP53* is the most frequently mutated genes in colon cancer (50%), while in rectal cancer it represents is second (60%) after adenomatous polyposis coli (*APC*). TP53 protein has a key role in cell cycle control, being responsible for G1-cell cycle arrest in order to allow for DNA repair before cell division. If DNA repair fails, *TP53* induces cell death through apoptosis. Similar to *KRAS*, *TP53* is targeted by miR-622. Studies evaluating miR-622 expression in CRC patients, showed opposite results: Ma et al. reported its downregulation in nCRT responders [49], while Della Vittoria Scarpati et al. found miR-622 upregulation [48]. Furthermore, TargetScan analysis highlights that many miRNAs identified as nCRT predictors target *TP53* mutation (Table 2).

*APC* is mutated in 61% of rectal cancers and in 48% of colon cancers. The adenomatous polyposis coli (*APC*) gene is an important tumor suppressor gene in CRC carcinogenesis [107]. Several miRNAs (mir-142, mir-153, miR-223, miR-125, miR-144, miR-16, miR-29) target APC. In particular, miR-125a and miR-144 are upregulated [45,48], while miR-29b-2 shows downregulation [50] in nCRT responders.

*KRAS* represents the third most frequently mutated gene in CRC patients (31% in colon cancer and 35% in rectal cancer). *KRAS* is a member of the MAP kinase pathway and its mutations are responsible for uncontrolled cell proliferation [108]. Using the TargetScan database, we pointed out that *KRAS* is targeted by three different miRNAs identified as nCRT predictors: miR-223-5p, miR-622, and miR-1246. miR-223-5p [47] and miR-1246 showed upregulation in nCRT responders [23,45], suggesting that, in these cases, *KRAS* downregulation is responsible for decreased cell proliferation. Another study showed that *KRAS* is also targeted by let-7e, whose expression is upregulated in nCRT responders and downregulated in non-responders [50].

*TGFBR2* gene is mutated in colon cancer (20%) but shows no mutation in rectal cancer. The product of this gene is a transmembrane protein that binds TGF-beta, forming a heterodimeric complex with TGF-beta receptor type-1. The main task of this complex is the phosphorylation of proteins, which then enter the nucleus and regulate the transcription of genes related to cell proliferation and carcinogenesis. Using the TargetScan database, we pointed out that TGFBR2 is targeted by miR-21. Caramès et al. found that miR-21 is downregulated in nCRT responders [57].

*FBXW7* is involved in protein ubiquitination, an essential step in the protein target degradation performed by the proteasome. *FBXW7* shows a mutation in 12% of colon cancers and in 14% of rectal cancers. Among miRNAs targeting *FBXW7*, Hotchi et al. and Nakao et al. found that miR-223 was upregulated in responders to nCRT, while Hotchi et al. found that miR-92a was downregulated [45,47].

The protein encoded by *PIK3CA* is involved in the cell response to various growth factors. Indeed, it is involved in AKT1 activation upon stimulation by receptor tyrosine kinase ligands such as EGF, insulin, IGF1, VEGFA and PDGF. Moreover, *PIK3CA* is essential in endothelial cell migration during vascular development through *VEGFA* signalling, possibly by regulating RhoA activity. *PIK3CA* participates in cardiomyogenesis in embryonic stem cells through the AKT1 pathway and plays an important role in vasculogenesis in embryonic stem cells through PDK1 and the protein kinase C pathway. According to the Cosmic database, *PIK3CA* is mutated in 18% of colon cancers and in 10% of rectal cancers.

Similar percentages of mutations in CRC cancer are reported for *SMAD4.* The protein encoded by this gene is a tumor suppressor inhibiting epithelial cell proliferation. Furthermore, it has an inhibitory effect on tumor growth, reducing angiogenesis and increasing the hyperpermeability of the blood vessels. In CRC patients, the *SMAD4* mutation rate is 13% in colon cancer and 12% in rectal cancer. Among the different miRNAs targeting SMAD4, Findlay et al. and Drebber et al. found that miR-145 was upregulated in nCRT patients and Hotchi et al. showed that miR-142 expression was increased in these subjects [45,52,53].

*BRAF* encodes for a protein involved in the regulation of the MAP kinase/ERK signalling pathway that is involved in cell division and differentiation. According to the Cosmic database, this gene is mutated in 14% of colon cancers and in 3% of rectal cancers.

**Figure 2:**
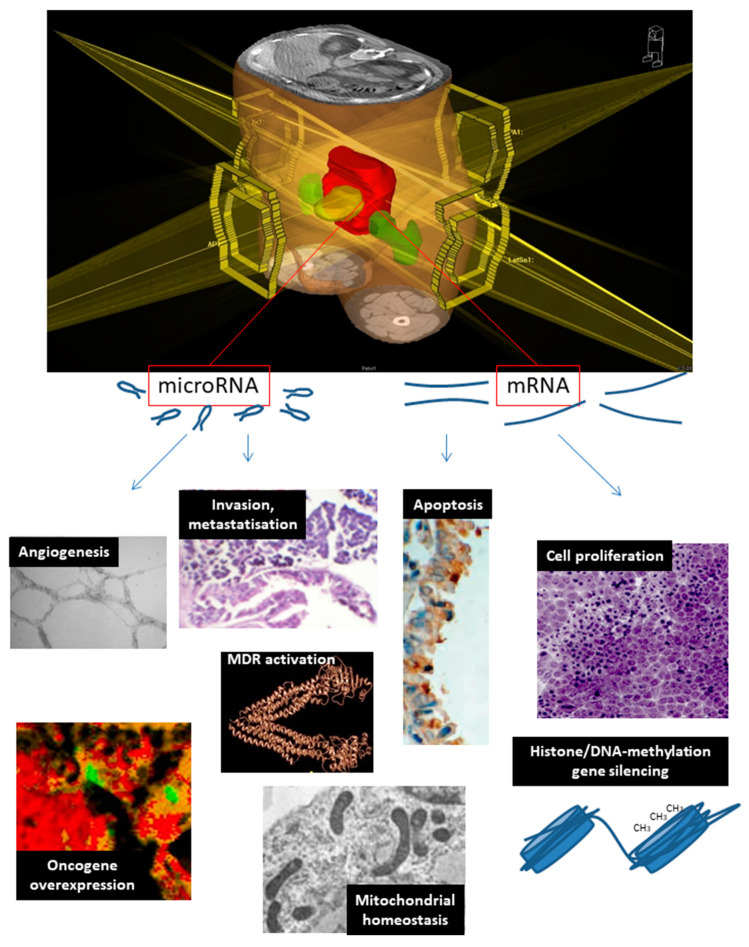
Biological functions of mRNA/miRNA predicting response of colon–rectal cancer to neoadjuvant therapy. The setting of radiation-based neoadjuvant therapy for colon–rectal cancer is shown in the upper panel, reporting converging radiation beams (yellow) and targeted cancer tissue (red). mRNA/miRNA response to neoadjuvant therapy, reflecting on biological processes (lower panels) and thus conditioning its efficacy.

An overall analysis of the ontology and main regulatory roles of mRNA and miRNA associated with nCRT response is reported in Figure 2 and Table 3.

The influence of mutations occurring in genes encoding for mRNAs associated with nCRT response, as inferred from The Cancer Genome Atlas – TCGA (https://www.cancer.gov/about-nci/organization/ccg/research/structural-genomics/tcga) is reported in Table 4.

## 5. Conclusions

The definition of the panel of biomarkers associated with treatment response is fundamental in order to predict nCRT effectiveness in CRC patients. Indeed, biomarkers are able to predict which patients will benefit from therapy. In addition, non-responder patients would avoid receiving an ineffective treatment.

This review has summarized the current knowledge on genetic and epigenetic biomarkers predictive of nCRT response. In most cases, these gene or miRNA signatures are particularly relevant and specific because, according to the inclusion criteria, they were identified in studies analysing CRC biopsies instead of blood.

Nevertheless, the heterogeneity among studies hampers the identification of a unique panel of biomarkers. Indeed, the study results are difficult to compare due to differences related to the following: (a) miRNAs and genes considered; (b) techniques used; (c) endpoints chosen; (d) tumor characteristics of patients enrolled; € protocol of nCRT administered; (f) cancer downstaging and downsizing criteria; (g) study design.

Despite these differences, the analysis of the scientific literature summarized in the present review may suggest, as a predictor of nCRT response, the miRNA signature composed of miR-145, miR-21, miR-622, miR-1183; miR-630, miR-1246 and miR-1290. These miRNAs have been identified according to the following criteria: (a) reported as associated with nCRT in two or more studies; (b) relationship with mutations targeting oncogenes playing a pathogenic role in CRC; (c) relationship with proteins whose expressions are altered in CRC. Furthermore, to be predictors of nCRT response, these miRNAs are biologically active biomarkers and could be considered in future studies. Retrospective studies should be replicated on large independent samples by means of a systematic approach, using homogeneous and clinically meaningful endpoints. Prospective studies are warranted to assess the clinical utility of the most promising biomarkers, aimed at overcoming nCRT resistance and improving patients’ response to therapy.

The management of CRC is very complex and thus it is very important to discover any factors that can predict the effectiveness of the treatments. This systematic review identifies miRNA and mRNA signatures that are potentially predictive of nCRT response. However, the wide heterogeneity of the selected neoadjuvant protocols, as well as the design of the studies and endpoints used, suggest that clinical studies are necessary to guide the application of this molecular signature in medical practice.

## Figures and Tables

**Table 1:** Top miRNAs predictors of neoadjuvant chemoradiotherapy (nCRT) response. Targeted genes were identified with TargetScan database. Only miRNAs for whom target mRNA were found in included articles are reported.

Top miRNAs	Target mRNAs	Gene Name	Total Context Score
miR-145	*TRIAP1*	TP53 regulated inhibitor of apoptosis 1	0.17
	*EGFR*	epidermal growth factor receptor	0.30
	*BAK1*	BCL2-antagonist/killer 1	0.20
miR-223	*KRAS*	Kirsten rat sarcoma viral oncogene homolog	0.09
miR-622	*TP53*	tumor protein p53	0.33
	*KRAS*	Kirsten rat sarcoma viral oncogene homolog	0.24
	*HIF1A*	Hypoxia-inducible factor 1, alpha subunit	0.17
	*VEGFA*	vascular endothelial growth factor A	0.16
	*EGFR*	epidermal growth factor receptor	0.07
	*MKI67*	antigen identified by monoclonal antibody Ki-67	0.10
	*HIF1A*	hypoxia-inducible factor 1, alpha subunit	0.20
miR-1246	*KRAS*	Kirsten rat sarcoma viral oncogene homolog	0.18

**Table 2:** Relationship between mRNA, miRNA and common oncogene mutations occurring in colorectal cancer. Common oncogene mutations were analysed using the Cosmic catalogue.

Protein	mRNA	miRNA
ABLIM1		miR-153; miR-335
ALDH3A1		miR-145
AMDHD2		miR-483; miR-1224
ARID1B		let-7a; miR-92a; miR-144; miR-363
ASAH1		miR-92a; miR-335
B3GALT5		miR-622
CBX1		miR-17; miR-20a; miR-20b; miR-106a; miR-223; mir-590
CDK	*CDK5R1*	miR-92a; miR-196b; miR-363
CDK	*CDKN1(A)*	let-7a; let-7g; let-7e; mir-16; miR-17; miR-20a; miR-106a; miR-125a; miR-145; miR-335; miR-363; miR-450a; miR-486; miR-542; miR-1909
CD177		miR-335
CNNM4		miR-92a; miR-363; miR-450b; miR-765; miR-1224
DPYD		miR-494
ENSA		let-7e; miR-1224
ERBB2		miR-21; miR-125a; miR-193a; miR-205; miR-486
FASTKD2		miR-16
FTO		miR-450a
glucose-6-phosphate 1-dehydrogenase		miR-335
GTF2E1		mir-31; mir-92a; mir-363; miR-561
IFIT1		miR-126; miR-335
LDH-A		miR-34b; miR-190b; miR-450b
LSM12		miR-561
MCMBP		miR-31; miR-154
PIP4K2B		miR-16; miR-215
PVR		miR-16; miR-17; miR-20a; miR-20b; miR-106a; miR-142; miR-519c
PVRL1		miR-765
RB1		miR-17; miR-20a; miR-20b; miR-21; miR-99a; miR-106a; miR-99a; miR-144; miR-215; miR-335; miR-450b; miR-494; miR-519c; miR-590; miR-622
SLC25A33		miR-17; miR-20a; miR-20b; miR-92a; miR-106a
SLC5A6		miR-379; let-7a; let-7e; let-7g
TRMT5		miR-20a; miR-153; miR-205
TSPAN6		miR-16; miR-17; miR-20a; miR-20b; miR-106a; miR-142; miR-144; miR-145
TYMP		miR-92a

**Table 3:** Gene ontology and main regulatory roles of mRNA and miRNA associated with neoadjuvant rectal CRT response.

Ontology	mRNA	miRNA	Reference
Mitochondrial function, CRC genetic susceptibility	*MT-ND4*, *MT-ND6*, *CDV3*		[109,110,111,112]
Histone gene silencing, signal transduction	*ITPK1*, *STK11*		[113,114]
Oncogene overexpression	*LGR5 (c-myc)*, *MYC*	let-7f (*K-RAS*), miR-21 (*PTEN*), miR-622 (*k-RAS*)	[115]
Invasion and metastatization	*NME2*, *RRM1*	miR-1183	[116,117,118]
Cell proliferation	*EGFR*, *RRM1*, *STK11*	let-7f, miR-21, miR-622, miR-630, miR-1183	[21,22,116,117,119,120]
Multidrug resistance	*KCNJ2*, *RRM1*	miR-630, miR-1183	[117,119,120]
Angiogenesis	*TYMP*, *VEGFA*	let-7f, miR-145	[30,31,32,33,34,40]
DNA methylation	*TYMS*		[35,36,37,38,39]
Apoptosis inhibition	*BIRC5*	miR-630	[16,17,18,120]

**Table 4:** TCGA database results of genes mutated in colon–rectal cancer encoding for mRNAs associated with neoadjuvant rectal CRT response.

Gene	% of Cases Affected	Gain of Function (%)	Loss of Function (%)	Survival in Mutation Carriers vs. Wild Type
***EGFR***	14	8	1	=
***LGR5***	9	1	5	Increased
***MYC***	8	23	0	Decreased
***VEGFA***	7	9	0	=
***STK11***	7	2	14	=
***TYMS***	4	7	2	Increased
***ITPK1***	4	1	6	Decreased
***BIRC5***	4	9	0	=

## Supplementary Materials

The following are available online at https://www.mdpi.com/2072-6694/12/6/1652/s1, Table S1: Overview of the full review strategy with search strings. Table S2: List of mRNAs differentially expressed in responders versus non responders. Table S3: List of miRNAs differentially expressed in responders versus non responders.
